# Analysis of liver miRNA in Hu sheep with different residual feed intake

**DOI:** 10.3389/fgene.2023.1113411

**Published:** 2023-10-19

**Authors:** Changchun Lin, Weimin Wang, Deyin Zhang, Kai Huang, Yukun Zhang, Xiaolong Li, Yuan Zhao, Liming Zhao, Jianghui Wang, Bubo Zhou, Jiangbo Cheng, Dan Xu, Wenxin Li, Xiaoxue Zhang, Wenxin Zheng

**Affiliations:** ^1^ College of Animal Science and Technology, Gansu Agricultural University, Lanzhou, Gansu, China; ^2^ Institute of Animal Husbandry Quality Standards, Xinjiang Academy of Animal Sciences, Urumqi, Xinjiang, China; ^3^ The State Key Laboratory of Grassland Agro-ecosystems, College of Pastoral Agriculture Science and Technology, Lanzhou University, Lanzhou, Gansu, China

**Keywords:** miRNA, residual feed intake, gene interactions, liver, sheep

## Abstract

Feed efficiency (FE), an important economic trait in sheep production, is indirectly assessed by residual feed intake (RFI). However, RFI in sheep is varied, and the molecular processes that regulate RFI are unclear. It is thus vital to investigate the molecular mechanism of RFI to developing a feed-efficient sheep. The miRNA-sequencing (RNA-Seq) was utilized to investigate miRNAs in liver tissue of 6 out of 137 sheep with extreme RFI phenotypic values. In these animals, as a typical metric of FE, RFI was used to distinguish differentially expressed miRNAs (DE_miRNAs) between animals with high (*n* = 3) and low (*n* = 3) phenotypic values. A total of 247 miRNAs were discovered in sheep, with four differentially expressed miRNAs (DE_miRNAs) detected. Among these DE_miRNAs, three were found to be upregulated and one was downregulated in animals with low residual feed intake (Low_RFI) compared to those with high residual feed intake (High_RFI). The target genes of DE_miRNAs were primarily associated with metabolic processes and biosynthetic process regulation. Furthermore, they were also considerably enriched in the FE related to glycolysis, protein synthesis and degradation, and amino acid biosynthesis pathways. Six genes were identified by co-expression analysis of DE_miRNAs target with DE_mRNAs. These results provide a theoretical basis for us to understand the sheep liver miRNAs in RFI molecular regulation.

## 1 Introduction

Feed efficiency (FE), an important economic trait in sheep production, is indirectly assessed by residual feed intake (RFI) and feed conversion ratio (FCR) (Carberry et al., 2012; Zhang et al., 2017a; Claffey et al., 2018; McGovern et al., 2018). RFI is defined as the discrepancy between the amount of feed actually consumed and amount anticipated to be needed for maintenance and growth (Mebratie et al., 2019). Improved FE has the potential to reduce meat production costs, with feed and feeding-related expenses accounting for 75% of total variable production costs in beef cattle farming (Ahola and Hill, 2012). Zhang et al. (2017b) showed in indoor sheep husbandry, feed expenditures account for 65%–70% of overall costs. In addition, research has been shown that enhancing ruminant FE may effectively mitigate greenhouse gas emissions and provide positive environmental outcomes (Nkrumah et al., 2006; Hegarty et al., 2007; Deng et al., 2018). Thus, livestock producers have a keen interested in the domain of genetic selection and breeding, particularly with regard to enhancing FE of their animals. However, the precise definition of FE in animals is currently being disputed, due to imperfect quality of ratios such as FCR (Gunsett, 1984). Therefore, RFI serves only as a metric for evaluating FE within the context of animal production. RFI is influenced by a variety of internal and external environmental variables such as body composition, nutrition digestion and metabolism, energy expenditure, physical activity, and control of body temperature (Zhang et al., 2017b). Recently, there has been a growing interest in the subject of FE within the context of livestock and poultry production, and researches on RFI-related genes have mainly focused on *swine* (Do et al., 2014; Jing et al., 2015; Horodyska et al., 2017; Messad et al., 2019), cattle (Santana et al., 2014; Salleh et al., 2018) and poultry (Yi et al., 2015). Animals with low_RFI exhibit reduced feed intake, and resulting in decreasedless waste and generation, which do not affect the body size, productivity, or weight of the animals (Koch et al., 1963). Therefore, studying the mechanisms of Low_RFI animals will not only reduce costs but also benefit the environment. The liver, being a vital digestive gland and metabolic organ (Xing et al., 2019), plays an important part in the metabolism of lipids, carbohydrates, and glucose metabolism, and has crucial physiological roles in oxidation, metabolism and reduction (El-Badawy et al., 2019; Cigrovski Berkovic et al., 2020; Ndiaye et al., 2020). Given the essential function played by the liver in the metabolic processes of livestock and poultry, it was selected as the sample in this present research.

MicroRNAs (miRNAs) are a class of small (∼22 nucleotides) endogenous non-coding RNAs that exhibit a high degree of conservation across different species (Nelson et al., 2011). The miRNAs have been discovered in several physiological fluids, tissues, and cell types, where they play a crucial role in regulating gene expression at the post-transcriptional level, and they are associated with a wide range of important biological processes (Halushka et al., 2018). Previous studies has shown that miRNA exert control over gene expression by their binding to particular messenger RNA (mRNA), which ultimately leads to the subsequent destruction or inhibition of the targeted transcript. miRNA is associated with the regulation of almost all cellular and developmental processes in eukaryotes (Nejad et al., 2018). For instance, it has been shown that miR-1, miR-133a, miR-133b, and miR-206 exhibit increased expression throughout the advanced phases of human of human fetal muscle development (Koutsoulidou et al., 2011). The miR-33 has inhibitory effects on the process of fatty acid breakdown by targeting several genes involved in fatty acid *β*-oxidation (Gerin et al., 2010). Moreover, miRNAs has been demonstrated to be essential for the development of brain structures and to support critical systems that, if disturbed, may lead to or cause neurodevelopmental disorders (Hollins et al., 2014). Previous studies have shown that a number of miRNAs in the liver tissues of various livestock play an important role in influencing FE. For instance, miR-338 influences fatty acid synthase (Xing et al., 2019), miR-185 affects glucose and lipid metabolism (Li et al., 2016), and miR-545-3p in pig liver affects fat deposition (Chu et al., 2017). In the liver of cattle, miR-19b regulates lipid metabolism of fat, miR-122-3p influences hepatic cholesterol and lipid metabolism, and miR-143 affects insulin signaling and glucose homeostasis (Al-Husseini et al., 2016). However, the precise processes via which miRNAs regulate RFI in sheep have yet to be fully unclear.

Thus, the present study aimed to identify candidate miRNAs that regulate FE to breed a Low_RFI Hu sheep population. We used sequencing to determine transcription differences in liver tissue of sheep with extreme RFI phenotypes.

## 2 Materials and methods

### 2.1 Ethical statement

The animal studies were done in accordance with the regulations and guidelines set out by the government of Gansu Province, as well as with the approval of the Animal Health and Ethics Committee of Gansu Agricultural University (Animal Experimentation License No. 2012-2-159).

### 2.2 Experimental animals and daily management

The experimental animals used in this study have been comprehensively detailed, together with their corresponding management regimens, in previous publications (Zhang et al., 2017b; Zhang et al., 2019). To put it simply, a total of 137 male Hu sheep were obtained from Jinchang Zhongtian Sheep Co., Ltd. (Jinchang China) and transported to Minqin Zhongtian Sheep Co., Ltd. (Minqin China) during the same time frame for the purpose of breeding. The process of weaning was established when the lambs reached 56 days of age. Subsequently, during the initial phases of the experimental study, only lambs displaying resilient development and overall outstanding health were selected as candidates. The lambs were supplied with nourishment in a standardized single pen (0.8 × 1 m), whereby they access to fresh water and food every day until the end of the experiment (180 days of age). The lambs attained an ideal age of 80 days for the start of the official experiment, which was recorded as day one. The performance experiment is concluded when the lambs reach 180 days, hence rendering the official duration of the trial as 100 days. Consequently, all lambs participating in this study had a 2-week transitional period prior before a 10 days pre-feeding phase. Throughout the transitional phase, a regular percentage adjustment was made to the form of feed utilized each day. Additionally, the whole pelleted feed was consumed on the initial day of the pre-fed phase. During the first 10-day pre-fed period and ensuing 100-day official trial, the pellet feed was purchased from Gansu Sanyang Jinyuan Animal Husbandry Co., Ltd., (Gansu China).

### 2.3 Phenotypic measurements and RFI calculation methods

Lambs were treated to a feeding regimen structured in 20-day cycles until the completion of the feeding experiment (180 days of age), with their initial weight being measured on the first day of the specified period (80 days of age). The lambs underwent daily weighing before to feeding, using a calibrated electronic scale. No modifications were made to the participants or the equipment utilized over the whole period of the experiment. Furthermore, each sheep was weighed for remaining feed before each weighing period, which was used to calculate feed intake and RFI. For the computational model used in this study, the main reference was the formula of Zhang et al. (2017b). The specific formula used in this particular instance was as follows:

$$Y_{k}=\beta_{0}+\beta_{1}{\text{MBW}}_{k}+\beta_{2}{\text{ADG}}_{k}+e_{k},$$

$$\text{MBW}={\left[\left({\text{BW}}_{i}+{\text{BW}}_{f}\right)/2\right]}^{0.75},$$

$$\text{ADG}=\left({\text{BW}}_{f}‐{\text{BW}}_{i}\right)/N,$$

where Y_k_ represents the average daily feed intake of the *i*th individual; β_0_ regres-sion intercept; β_1_ regression coefficient for mid-test metabolic body weight (MBW); β_2_ regression coefficient for average daily gain (ADG); e_k_ represents uncontrolled error of the *i*th individual; BW_f_ represents final body weight; BW_i_ represents initial body weight; and N, experimental period (days).

### 2.4 Liver tissue collection and total RNA extraction

The methodologies used for the collection and processing methods of the tissues were cited from previous scholarly investigations (Zhang et al., 2017b). The methodology may be concisely summarized in the following manner: all lambs are uniformly transferred to a professional slaughterhouse after the end of the measurement. The RFI values of the six sheep used in this study are provided fully, concerning prior research conducted by our research group. The lambs were subjected to a 24 h of fasting period before to being weighed and then executed in standard procedures. Each liver sample was immediately collected after slaughter process and then preserved in liquid nitrogen for temporary storage. Following the process of slaughter, it was transferred to −80°C for long-term storage until RNA was extracted. As described in previous study, we selected 3 High_RFI and 3 Low_RFI sheep from 137 male Hu lambs for total RNA extraction (Zhang et al., 2017b; Zhang et al., 2019). The total RNA extraction was performed using the TRIzol Reagent (Invitrogen, Waltham, MA, United States) method as per the provided instructions. The NanoPhotometer^®^ spectrophotometer (IMPLEN, CA, United States) was used to quantify the purity of RNA, while the integrity of RNA was performed using the Agilent Bioanalyzer 2,100 system’s RNA Nano 6000 Assay Kit (Agilent Technologies, CA, United States).

### 2.5 Library preparation and small RNA sequencing

The small RNA library preparation kits were used to produce small RNA sequencing libraries (Galina-Pantoja et al., 2006). There are many steps that involved, as seen below: firstly, whole RNA molecule was used as a template to directly connect the 3′ and 5′ ends of the small RNA with the synthetic adaptors. The synthesis cDNA from total RNA was conducted with M-MuLV reverse transcriptase (NEB, United States), followed by amplification of the resulting cDNA in accordance with the recommended protocols provided by Illumina. The PCR products underwent purification and recovery processes using 8% polyacrylamide gels. These gels exhibited the capability to effectively separate DNA fragments with sizes 140 to 160 base pairs. Following that, the DNA fragments that had undergone purification were dissolved in 8 μL of elution solution. In the end, the evaluation of the library’s integrity on the Agilent Bioanalyzer 2,100 system may be conducted by using DNA high-sensitivity chip. Once the library has undergone qualification, the product was subjected to sequencing on the Illumina HiSeq 2,500 platform, resulting in the generation of a 50 bp single-ended read.

### 2.6 Bioinformatics sequence data processing and miRNA expression profiling

The raw data collected by the sequencing equipment was then given a Base Calling analysis with the intention of producing FASTQ files. The acquisition of clean data included the elimination of extraneous information from the raw data via the use of a customized Perl script. Concurrently, Q20, Q30, and GC-contents of the raw data were obtained. Then, for all subsequent analysis, choose certain range of length from clean reads. The Bowtie (Langmead et al., 2009) method was used for the purpose of aligning small RNA tags with the reference sequence, facilitating the assessment of their expression levels on the reference. Then used miRbase 20.0 as a reference to mapping known miRNAs. In order to exclude protein-coding genes, repetitive sequences, rRNA, tRNA, snRNA, and snoRNA, custom scripts are used to extract miRNAs of predetermined length. Following, the software tools miREvo (Wen et al., 2012) and mirdeep2 (Friedländer et al., 2012) were used to map sequences with the sheep reference genome (*Oar_v1.0*) to provide predictions about novel miRNAs. The use of the whole rRNA ratio served as an indicator of sample quality. In animal samples, this ratio should ideally be below 40%. Additionally, the cumulative *p* values for RNA folding were used as a metric for output measure.

### 2.7 Differential expression analysis and prediction of target genes of miRNAs

The miRNA expression levels were estimated by TPM (transcript per million) (Zhou et al., 2010). The processing procedure differs according on the sample’s quality. To perform differential expression analysis on Low_RFI and High_RFI samples with biological recurrence, use the DESeq R package (version 1.8.3). The filtering conditions for differential expression of miRNAs in this study were *p*-value <0.05 and |log2 (foldchange)|≤ 0.5 (Tang et al., 2007). The threshold for substantial differential expression is set to the default value. The target gene of miRNA was then predicted for animals using miRanda (Enright et al., 2003). Statistics calculations were carried out to analyze the expression levels of the most prevalent known miRNAs and novel miRNAs in both experimental groups. Additionally, the most common miRNA or DE_miRNA expression was estimated in relation to the anticipated mRNA expression of its target gene.

### 2.8 GO and KEGG enrichment analysis

The target gene candidates of DE_miRNAs were analyzed using Gene Ontology (GO) enrichment analysis (“target gene candidates” in the follows). For GO enrichment analysis, a GOseq-based Wallenius non-central hyper-geometric distribution (Young et al., 2010) was used, which might correct for gene length bias. Kyoto Encyclopedia of Genes and Genomes (KEGG) (Kanehisa et al., 2008) was a database used to understand the advanced functions and benefits of biological systems based on molecular-level information, especially genome sequencing and other highly experimental technologies (http://www.genome.jp/kegg/). To assess the statistical enrichment of target gene candidates in KEGG pathways, we employed the KOBAS (Mao et al., 2005) tool.

### 2.9 Integral miRNA–mRNA networks analysis

Investigate the possible association between DE_miRNA found in this study and Zhang et al. describes DE_mRNA (Cigrovski Berkovic et al., 2020). The construction of a miRNA-mRNA interaction network was facilitated by using Cytoscape software (Shannon et al., 2003). This network was established by including DE_miRNAs and DE_mRNAs based on their specific roles. Specifically, mRNAs exhibiting discernible association with miRNAs were integrated into the miRNA-mRNA interaction network. The same shape with various colors shows types that are up-or downregulated in DE_miRNAs and DE_mRNAs.

## 3 Results

### 3.1 miRNA sequence data and mapping quality

**FIGURE 1:**
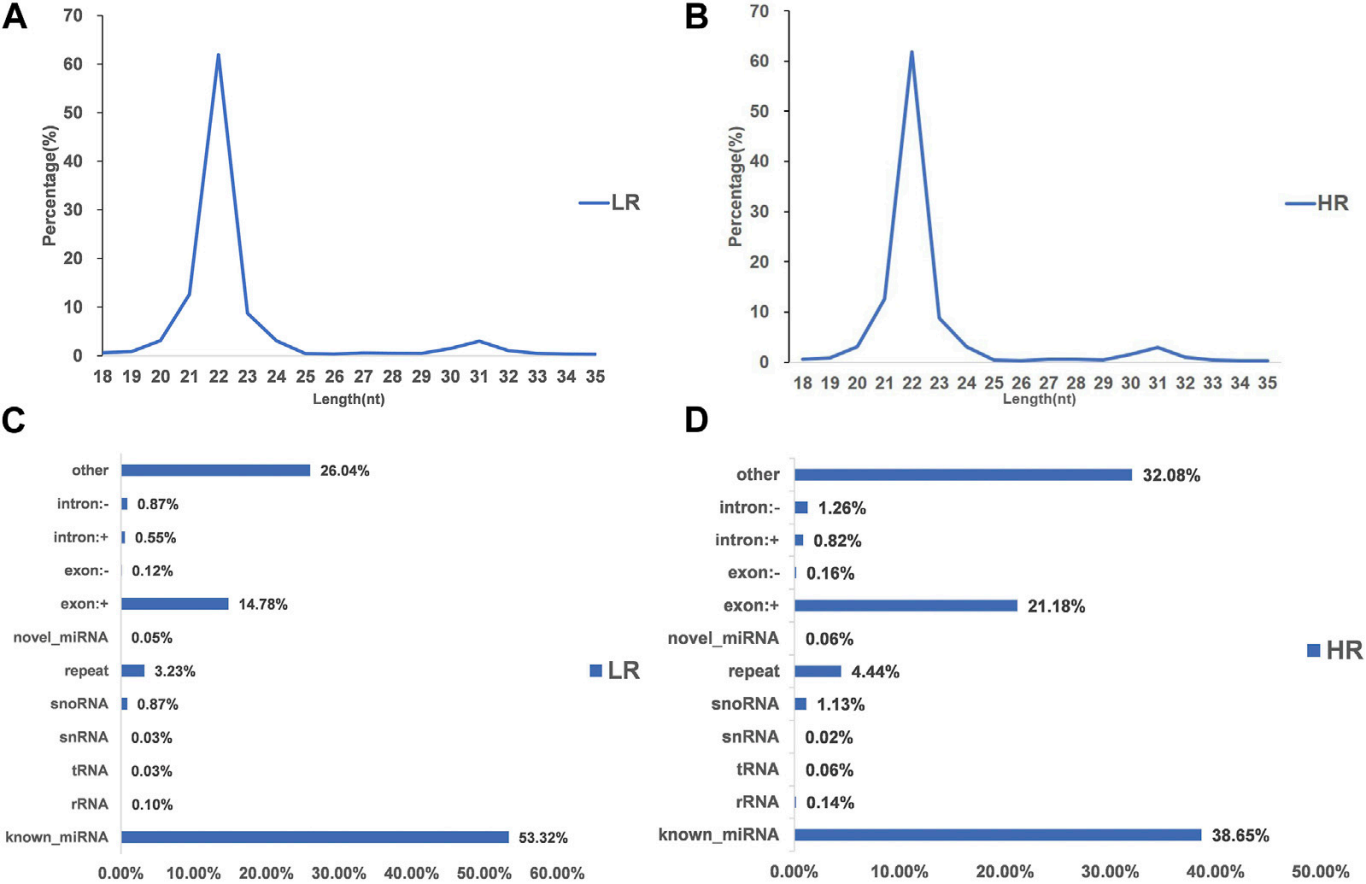
Characterization of microRNA (miRNA) profiling and the percentage of detected miRNAs from ncRNAs. **(A, B)** Length distribution of clean reads from identified miRNA fragments. **(C, D)** Categories of identified non-coding RNAs (ncRNAs) via sequencing in Low_FRI and High_FRI. Note “LR: Low_RFI, HR: High_RFI” (the following figures are identical).

3.2 Illumina sequencing generated more than 10 M (million) high quality raw reads for each of the two groups of Hu sheep (except for High_RFI No.1 Hu sheep) (Table 1). The raw data obtained from sequencing is given to a filtering process so as to generate clean reads. Reads with more than 10% N content were removed first. An average of 124 reads was removed in the Low_RFI group, less than 0.01%, and similarly an average of 133 reads was removed in the High_RFI group, less than 0.01% (Supplementary Table S1). Further, after removing low-quality readings, the Low_RFI group averaged 0.32% and the High_RFI group 0.35% being removed (Supplementary Table S1). The number of deletions resulting from the existence of 5 adapter contamination and the presence of ployA/T/G/C was low, with an average 0.00% and 0.03% in the Low_RFI group 0.00% and 0.06% in the High_RFI group, respectively (Supplementary Table S1). More reads were deleted due to the absence of 3 adapter null or insert null, about 0.97% and 0.95%. All samples were filtered to retain clean reads of 98% or more. Finally, the clean reads of each sample were screened for sRNAs within a certain length range (18∼35 nt) for subsequent analysis (Table 1). The length-screened sRNAs were localized to the *Ovis aries* reference genome to analyze the distribution of small RNAs (Table 1). From the clean data, a total of 10,278,758, 10,014,172, 9,533,396, 8,699,934, 9,391,798, and 13,765,901 mapped reads from the LR1 (Low_RFI No.1 Hu sheep), LR2 (Low_RFI No.2 Hu sheep), LR3 (Low_RFI No.3 Hu sheep), HR1 (High_RFI No.1 Hu sheep), HR2 (High_RFI No.2 Hu sheep), and HR3 (High_RFI No.3 Hu sheep) libraries were retrieved, with over 90% mapping to the *Ovis aries* reference genome (Table 1). In terms of miRNA expression level, our research results indicate that genes with a TPM <60 retrieved from RNA-seq accounted for about 75% of the total, whereas high-expressed genes, that is, genes with TPM >60, accounted for around 25% (Table 2). However, HR2 accounted for only 7.81%. Screening miRNAs ranged from 18 to 35 nt in length (Figures 1A, B). Furthermore, a variety of non-coding RNAs (ncRNAs) were discovered, including transfer RNAs (tRNAs), snRNAs and miRNAs (Figures 1C, D). Among the identified ncRNAs, a minute fraction constituted recently discovered miRNAs.

**TABLE 1:** Summary of clean reads mapped to the *Ovis aries* reference genome.

Sample	LR1	LR2	LR3	HR1	HR2	HR3
Raw Reads	11,585,179	10,414,448	11,147,151	9,959,923	10,728,970	15,885,959
Clean Reads	11,120,503	10,206,637	10,509,901	9,583,661	10,176,841	15,142,493
Q30 (%)	97.98	98.62	97.82	97.97	97.83	97.81
GC Content (%)	48.91	48.36	49.19	49.19	48.92	48.99
Raw Reads	11,585,179	10,414,448	11,147,151	9,959,923	10,728,970	15,885,959
Total Mapped	10,278,758 (92.43%)	10,014,172 (98.11%)	9,533,396 (90.71%)	8,699,934 (90.78%)	9,391,798 (92.29%)	13,765,901 (90.91%)

Notes: LR1: Low_RFI, No. 1 Hu sheep; LR2: Low_RFI, No. 2 Hu sheep; LR3: Low_RFI, No. 3 Hu sheep; HR1: High_RFI, No. 1 Hu sheep: HR2: High_RFI, No. 2 Hu sheep; HR3: High_RFI, No. 3 Hu sheep; Q30: (Percentage of bases with phred values greater than 30 in the total number of bases); GC, Content: Calculate the sum of the number of bases G and C as a percentage of the overall number of bases.

**TABLE 2:** Analysis of miRNA expression levels.

Sample	LR1	LR2	LR3	HR1	HR2	HR3
0–0.1	62 (24.22%)	46 (17.97%)	37 (14.45%)	58 (22.66%)	145 (56.64%)	38 (14.84%)
0.1–0.3	35 (13.67%)	36 (14.06%)	24 (9.38%)	25 (9.77%)	29 (11.33%)	0 (0.00%)
0.3–3.57	55 (21.48%)	66 (25.78%)	85 (33.20%)	67 (26.17%)	36 (14.06%)	99 (38.67%)
3.57–15	30 (11.72%)	28 (10.94%)	26 (10.16%)	32 (12.50%)	16 (6.25%)	28 (10.94%)
15–60	17 (6.64%)	21 (8.20%)	23 (8.98%)	18 (7.03%)	10 (3.91%)	24 (9.38%)
>60	57 (22.27%)	59 (23.05%)	61 (23.83%)	56 (21.88%)	20 (7.81%)	67 (26.17%)

### 3.2 Known miRNA expression and novel miRNA profiles

We identified 121, 120, and 122 known miRNAs in the High_RFI samples, and 119, 83, and 128 known miRNAs in the Low_RFI samples (Table 3). In all of these reads, approximately 59% of known miRNAs were detected in all samples (Supplementary Table S2). The miRNA the highest abundance across all samples was oar-miR-148a, with mean paired reads from HR1, HR2, HR3, LR1, LR2, and LR3 of 2,885,255, 1,771,013, 2,178,809, 3,049,001, 7,921,051, and 1,323,994, respectively. Among the top 20 expressed miRNAs in each group, the expression levels of 10 miRNAs such as oar-miR-148a, oar-let-7f, oar-miR-143, oar-miR-30a-5p, oar-miR-26a, oar-miR-21, oar-let-7g, oar-let-7i, oar-miR-30d and oar-miR-99a accounted for an average of more than 94% (Supplementary Table S3). The expression analysis shows the top 20 highly expressed miRNAs in the study samples from each respective group (Supplementary Figure S1).

**TABLE 3:** Number and ratio of identified miRNA matrices.

Types	Total	HR1	HR2	HR3	LR1	LR2	LR3
know	139	121	120	122	119	83	128
ratio	100%	100%	87%	86%	88%	86%	60%
novel	117	73	90	97	79	28	90
ratio	100%	62%	77%	83%	68%	24%	77%

The hairpin structure that is characteristic of miRNA precursors may be used as a means to forecast the existence of novel miRNAs. We identified 73, 90, and 97 novel miRNAs in the High_RFI samples, and 79, 28, and 90 novel miRNAs in the Low_RFI samples (Table 3). Among the novel that were identified, only 23 were identified in all samples (Supplementary Table S3). Of the 117 unique novel miRNAs, novel_31, novel_50 and novel_41were the most expressed in the samples with an average of 1,826, 1,246 and 1,173 reads aligned to these miRNAs, respectively (Supplementary Table S3). All detected new miRNAs were supplied (Supplementary Table S3), and the top 20 expressed novel miRNAs for each group were presented (Supplementary Table S4).

### 3.3 miRNA differential expression

**FIGURE 2:**
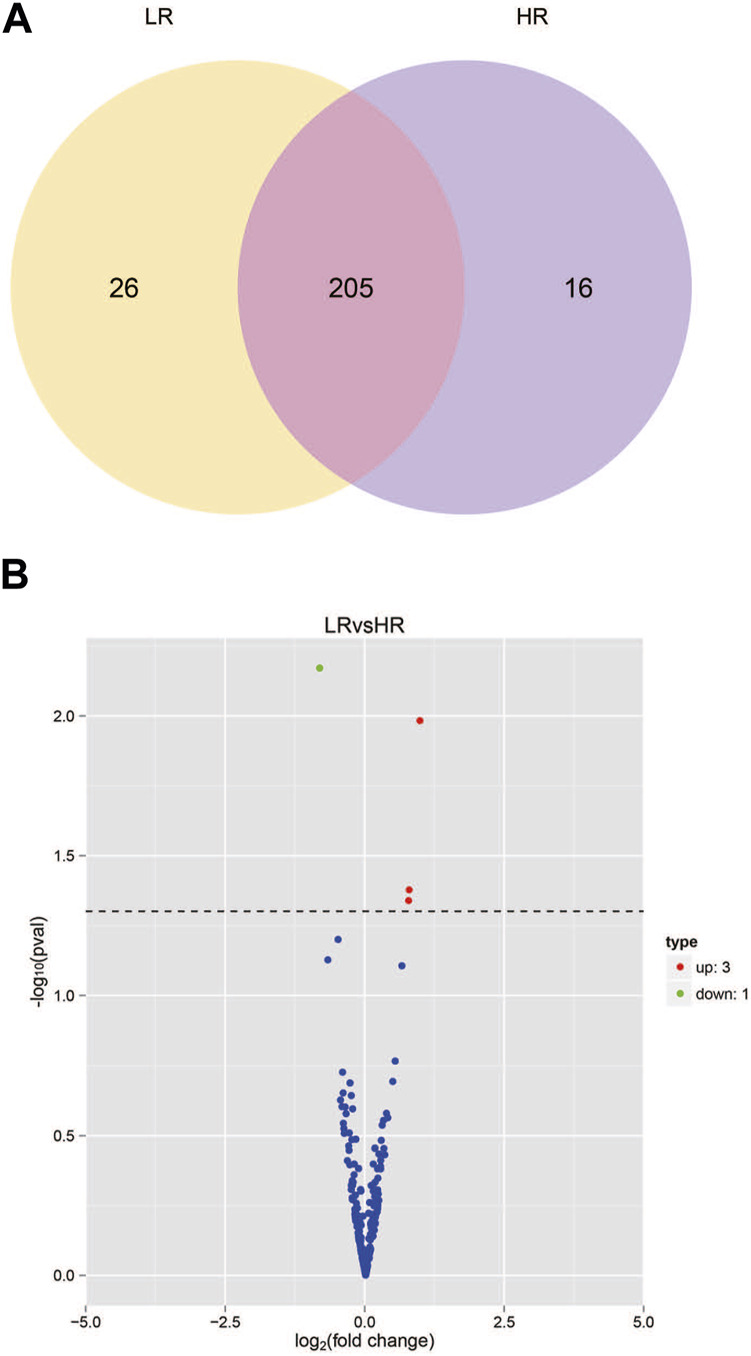
miRNA expression in the liver of sheep with different RFI. **(A)** Venn diagram showing the total number of miRNAs expressed in each group individually and in both. **(B)** Volcano map of differentially expressed miRNAs. In the volcano plot, significant downregulated genes are indicated by “green” dots, while significant upregulated genes are indicated by “red” dots.

The sequencing data has been submitted to the NCBI Sequence Read Archive (SRA) database under the biological project PRJNA813431. Differential miRNA expression analysis between Low_RFI and High_RFI groups from the same population of sheep with different phenotypic values. The relevant phenotypic data for the selected sheep were detailed in previous studies (Zhang et al., 2022). Phenotypic differences were not significant except for FCR, RFI and feed intake (FI). In total, an average of 11.62 million raw read were obtained from each sample. A total of 247 miRNAs were detected in 6 liver samples, of which four miRNAs (one known miRNA and three novel miRNAs) were identified as differentially expressed (*p* < 0.05) (Table 4). The Venn diagrams show miRNAs that are uniquely expressed or co-expressed in different groups, with 205 miRNAs co-expressed in both groups (Figure 2A). Four miRNAs were differentially expressed in the Low_RFI group compared to the High_RFI group, including three upregulated and a downregulated (*p* < 0.05) (Table 4) (Figure 2B). Of all the DE_miRNAs identified, novel_41 and novel_115 were expressed in all samples. The novel_41 was upregulated in Low_RFI group, while novel_115 showed downregulated compared to the High_RFI group (Table 4).

**TABLE 4:** Four differentially expressed miRNAs in Hu sheep with Low and High_RFI.

miRNA	log2FoldChange	*p*-value	Mature sequence
novel-171	0.97	0.0104	aau​cag​uau​cug​ucu​ggg​uag​a
novel-41	0.78	0.0421	uca​cug​ggc​auc​cuc​ugc​uuu
oar-miR-485-3p	0.77	0.0459	guc​aua​cac​ggc​ucu​ccu​cuc​u
novel-115	−0.82	0.0068	uug​cac​aac​ucu​aga​aga​cau​g

### 3.4 Target gene prediction and functional enrichment analyses for the most abundant known and novel miRNAs

The target genes of ten most highly expressed miRNAs (seven known and three novel) were predicted in the two groups for further functional analysis (Supplementary Table S5). The majority of these target genes mainly involved in various biological processes, including glycolysis, protein synthesis and catabolism, cell growth and proliferation, scavenging of free radicals, as well as cell death and survival (Supplementary Figure S2). In terms of glycolytic processes, the target genes were mainly involved in nucleoside diphosphate kinase activity, nucleoside kinase activity, adenylate kinase activity, hexokinase activity, and other glucose binding activities. For protein synthesis and catabolism, target genes were involved in the positive regulation of protein secretion, protein polymerization and protein deubiquitination, among other roles. For cell growth and proliferation, target genes were involved in platelet alpha granulation, cell cortex proliferation, and the proliferation and development of microtubule cell ribosomes (Supplementary Figure S2).

### 3.5 DE_miRNAs target gene prediction

**FIGURE 3:**
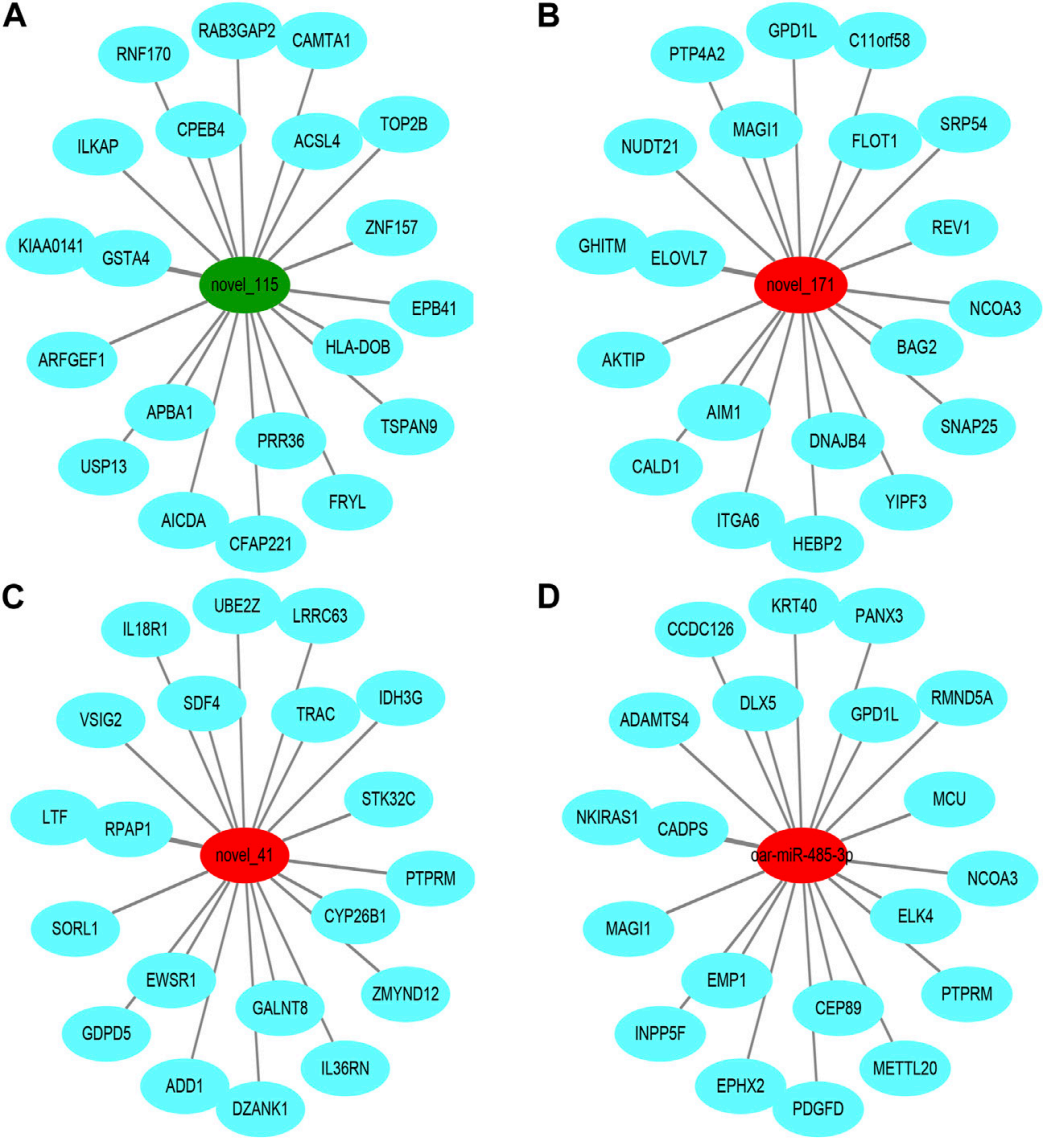
Top ranked (based on total target score of miRDB) DE_miRNAs for target genes. In the network plot, significant downregulated genes are indicated by green, while significant upregulated genes are indicated by red. The target gene predicted by psRobottar is shown in blue.

**FIGURE 4:**
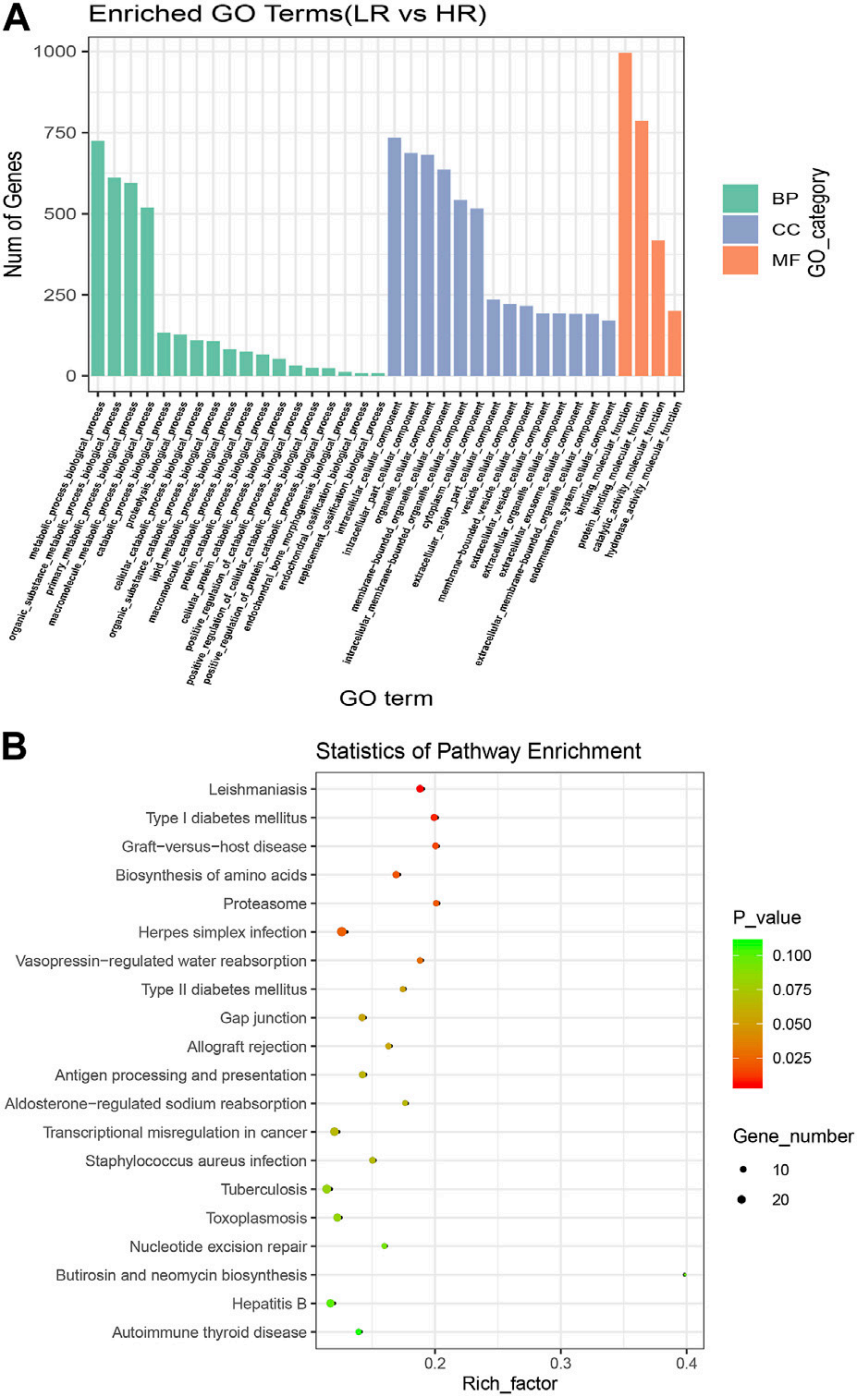
Enrichment analysis of high and Low_RFI differential miRNAs. **(A)** Using GO (Gene Ontology) enrichment analysis, BP indicates biological process, CC indicates cellular component, MF indicates molecular function. **(B)** Kyoto Encyclopedia of Genes and Genomes (KEGG) differentially expressed miRNAs target gene enrichment.

To further understand the biological functions and roles of these four DE_miRNAs, target genes were identified (miRDB) for highly differentiated miRNAs between Low_RFI and High_RFI gruops. Upregulated miRNAs in Low_RFI group were integrated to several target genes: top ranking genes were *DZANK1*, *CYP26B1*, *IDH3G*, *TRAC*, *IL36RN*, *UBE2Z*, *ZMYND12*, *PDGFD*, *VSIG2,* and *ELK4*, whereas top-ranking target genes with downregulated miRNAs were *CFAP221*, *HLA-DOB*, *TOP2B*, *ACSL4*, *FRYL*, *RAB3GAP2*, *TSPAN9*, *ILKAP*, *USP13,* and *PRR36*. The provided diagram illustrates the top 20 anticipated target genes for the four DE_miRNAs (Figure 3). To further elucidate the functions of the DE_miRNAs, we performed enrichment analysis of their candidate target genes. GO enrichment results showed that these target genes were mainly related to metabolism and binding: Biological processes: metabolism, organic metabolism, primary metabolism and macromolecular metabolism. Cellular components: intracellular, intracellular fractions, organelles and membrane-bound organelles. Molecular functions: binding, protein binding, catalytic activity and Hydrolytic enzyme activity (Figure 4A). It was shown that the metabolism in the liver plays an important role in the efficiency of animal feed. KEGG pathway showed DE_miRNAs target genes were mainly enriched in transcriptional dysregulation in herpes simplex virus infection, leishmaniasis, and biosynthesis of amino acids (Figure 4B).

### 3.6 Target gene matching between previously identified DE_mRNAs and the DE_miRNAs prediction

**FIGURE 5:**
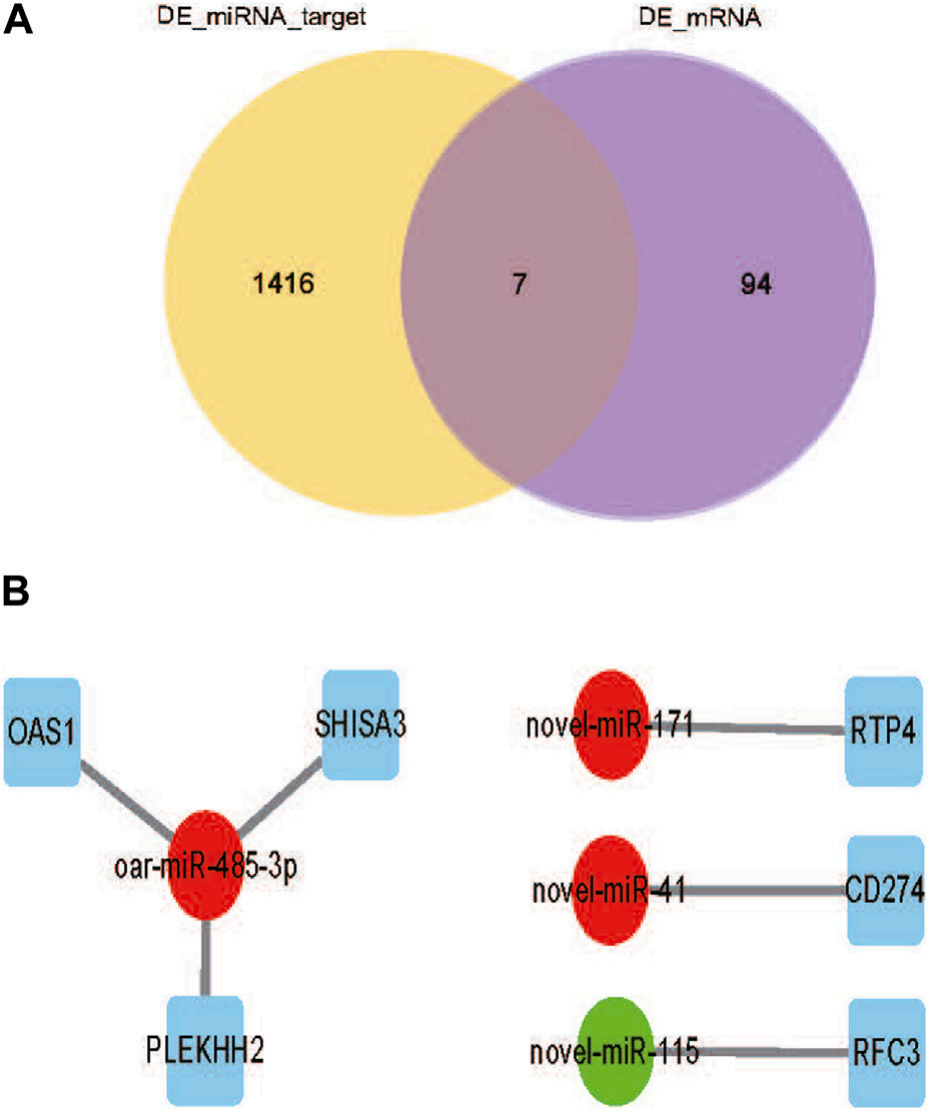
**(A)** Venn diagram showing the number of differential mRNA target genes in yellow, the number of differential miRNA candidate target genes in purple, and the overlapping part indicates the co-expressed part. **(B)** DE_miRNAs are indicated by circles, significant downregulated genes are indicated by green, while significant upregulated genes are indicated by red. And squares indicate DE_miRNAs co-expressing target genes with DE_mRNA.s.

To fully understand the potential RFI effects of miRNA, we used DE_miRNA and their targets genes to create an interactive population network. A total of 1423 DE_miRNAs target genes were identified. We previously discovered 101 DE_mRNAs between the low and high RFI sheep groups using the same objective as the present investigation (Zhang et al., 2022). Among these, seven miRNAs were co-expressed (Figure 5A). However, some target genes were predicted to be the target genes of a single miRNA, and it was observed that upregulation of DE_miRNAs did not always result in downregulation of DE_mRNAs in liver tissue obtained from animals with Low_RFI. Most DE_miRNAs were predicted to primarily target a single differential target gene. As shown in figure, the upregulated miRNA novel_171 targets the upregulated target gene *RTP4*, and the upregulated miRNA novel_41 targets the downregulated target gene *CD274* (Figure 5B). However, the upregulated miRNA oar-miR-485-3p targeted three different genes, the downregulated target gene *OAS1* and the two upregulated target genes *SHISA3* and *PLEKHH2*.

## 4 Discussion

RNA sequencing can serve as a powerful miRNAs expression profiling tool to identify the DE_miRNAs(Motameny et al., 2010), even at low expression levels in all cells, as well as allows for the parallel analysis of known miRNAs and the identification of miRNAs (Pritchard et al., 2012). Along with the simultaneous analysis of known miRNAs, the examination of novel miRNAs also becomes feasible. Furthermore, the use of mature miRNA sequences may facilitate the identification of prospective target genes for both known and undiscovered miRNAs. In this study, miRNAs sequencing was used to identify miRNA expression profiles in liver tissue from 6 Hu sheep with extreme RFI from the same farm. Sequencing results showed that sequencing data were of high quality with an average Q30 value of 94%. In addition, after quality control processing of raw sequencing data, the read sequences was an average length of 22 bp, while the majority of reads f were ranged between 20 and 24 bp length from both Low_RFI and High_RFI gruops, providing a high quality and reliable data for subsequent analysis (Friedländer et al., 2012) (Figures 1A, B). The observed average alignment rate, above 90%, indicates a strong agreement between the identified miRNAs and the liver samples. Furthermore, roughly 83% of these miRNAs were found to be expressed in all liver samples, which aligns with the findings of a previous research on miRNAs in bovine liver (Mukiibi et al., 2018). This observation implies that miRNAs exhibit a high degree of conservation within a given population. Among the miRNAs that have been identified, ten highly expressed miRNAs, including oar-miR-148a, oar-let-7f, oar-miR-143, oar-miR-30a-5p, oar-miR-26a, oar-miR-21, oar-let-7g, oar-let-7i, oar-miR-30d and oar-miR-99a, accounted for an average of 92.14% and 95.92% of the total aligned sequence reads in the High and Low_RFI groups, respectively. According to a publication, let-7 miRNA has been detected in various animal species, including humans (Lee et al., 2016). In the present study, it was shown that oar-let-7f, oar-let-7g and oar-let-7i which are members of the let-7 family in sheep, had highly expressed levels in the liver of both groups of FRI sheep. This finding suggests that let-7 family of miRNAs was substantially conserved. The description was consistent with the previously reported results (Friedman et al., 2009). Therefore, from this we speculate that let-7 family miRNAs have the same trend in the same species. Interestingly, oar-miR-148a was the most highly expressed miRNA in all samples, and it belongs to the miR-148/152 family, whose homologous members are involved in a variety of biological functions and diseases in different species. For example, it has been reported that overexpression of miR-148 significantly promotes myogenic differentiation in C2C12-derived myoblasts and primary myoblasts (Zhang et al., 2012). In sheep, miR-148a have been reported to accelerate lipogenic differentiation of sheep preadipocytes and inhibit the proliferation of sheep preadipocytes by inhibiting *PTEN* expression (Jin et al., 2021). Furthermore, it had an inhibitory effect on the proliferation of Hu sheep hair papilla cells and was associated with hair follicle growth and development (Lv et al., 2019).

To explore the biological significance of sheep-associated DE_miRNAs with varying RFI characteristics. We performed target gene prediction for ten miRNAs that were highly expressed in two groups of RFI sheep. Among these target genes, the main biological functions involved include: negative term regulation of the apoptotic process, cell growth and proliferation, apoptosis and survival, and adipocyte differentiation. Some miRNAs with higher abundance have been discovered as significant regulators of animal cell proliferation and development, apoptosis, and regeneration, which is consistent with our results (Wang et al., 2009). As an example, the second highest expressed miRNA in our research, namely, oar-miR-30a-5p, has been previously associated to lipid and insulin metabolism in mice (Sud et al., 2017; Kim et al., 2019). miR-26a and miR-143 are involved in the regulation of mouse hepatocyte proliferation, a significant aspect in liver tissue regeneration (Geng et al., 2016; Zhou et al., 2019). miR-99a and miR-148a (Gailhouste et al., 2013) were identified as regulators hepatic detoxification in liver tissues of mice and human animals. Based on the observed of miRNA-mRNA interactions across mammalian species and our results of our study, we hypothesized that these miRNAs highly expressed in sheep liver may perform similar biological functions to other species. Moreover, since these highly expressed miRNAs are in a state of continuous self-regeneration or regeneration, it explains their involvement in proliferation as well as apoptosis and regeneration of different cells.

The liver, being the biggest internal organ, plays crucial functions in several physiological metabolic processes, including detoxification (He et al., 2020; Wang et al., 2020). It also serves as a central regulator of energy metabolism, with glycation as a fundamental feature, and is an important coordinator of metabolism and a key site for maintaining metabolic homeostasis (He et al., 2017; Matz et al., 2017; Xue et al., 2019; Moscoso and Steer, 2020). It has been reported that miRNAs are involved in almost every aspect of cell biology (Chen and Verfaillie, 2014). miRNAs play crucial biological roles in cell differentiation, proliferation, metabolism and apoptosis, as well as in viral infection (Kim et al., 2009). Hence, the variable expression of liver miRNAs in Low_RFI and High_RFI sheep might potentially lead to molecular differences in FE. In this study, one known and three novel miRNAs were identified between Low_RFI and High_RFI gruops. However, most of the detected DE_miRNAs (50%) were conservative, which is consistent with the conclusion that miRNAs are conservative (Friedman et al., 2009). For this study to validate the present findings, it would be necessary to conduct more investigations including bigger cohorts of sheep and more broad range of phenotypic animal populations, given that a lower threshold of DE_miRNA screening (*p* < 0.05) was used. In the present study, more than 75% of the DE_miRNAs were upregulated in Low_RFI animals, which was consistent with the results of differential expression analysis of miRNAs in beef cattle with different FE phenotypes (Mukiibi et al., 2020). Thus, this suggests that reduced expression of target genes for these miRNAs is expected.

To investigate the potential biological role of RFI-associated DE_miRNAs in sheep, we predicted their target genes. The target genes are associated with many crucial biological activities, such as metabolic processes, organic metabolism, cell assembly and structure, lipid metabolism, protein breakdown, protein binding, protein metabolism, catalytic activity, and hydrolytic enzyme activity. Among these functions, lipid metabolism and protein synthesis have been reported to be relevant in other species (Chen et al., 2011; Alexandre et al., 2015; Tizioto et al., 2015; Mukiibi et al., 2018). To further understand how DE_miRNAs interact with the 101 DE_mRNAs identified in previous studies (Zhang et al., 2019). Only seven DE_mRNAs (annotated as *RTP4, CD274, OAS1, PLEKHH2, SHISA3*, and *RFC3*) were identified as target genes for DE_miRNAs. These target DE_mRNAs play important roles in innate antiviral response, protein-coupled receptor trafficking, immunity, cell mobility, intercellular signaling and connections, cell death, cell development, differentiation, and gene regulation. Meanwhile, the *RTP4* gene has been shown to be associated with RFI in sheep (Zhang et al., 2022). Certain DE_miRNAs have the potential to impact hepatic functional efficiency FE via their distinct regulatory effects on several biological processes inside the liver. According to the DE_miRNAs- mRNAs interaction network (Figure 5), some single miRNAs were predicted to be targets of single or multiple DE_mRNAs. Because a single miRNA using its seed region may bind to multiple sites in the 3′-UTR of distinct genes (mRNAs), and one target can have multiple binding sites for one or more miRNAs, miRNAs can modulate multiple biological processes even if they are few in number compared to mRNAs they regulate (Ambros, 2004; Bartel, 2004; Brennecke et al., 2005).

Overall, the comparison of DE_miRNAs and DE_mRNAs expression patterns in liver tissue that we identified was consistent with expectation. This may be attributed to the fact that miRNAs accelerate degradation of target genes by promoting the deadenylation of their target transcripts (Stroynowska-Czerwinska et al., 2014). Consequently, we have observe different patterns of DE_miRNA targeting of DE_mRNAs, perhaps attributable to variations in the regulatory mechanisms governing mRNA degradation. To better understand the relationship between miRNAs and mRNAs, further studies at the cellular level are needed to verify these interactions.

## 5 Conclusion

In the present study, we employed RNA-seq to analyze liver miRNAs in sheep populations. Among these miRNAs, oar-miR-148a, oar-let-7f, oar-miR-143, oar-miR-30a-5p, oar-miR-26a, oar-miR-21, oar-let-7g, oar-let-7i, oar-miR-30d and oar miR-99a had the highest expression levels in all samples. By differential miR-mRNA expression analysis, four miRNAs associated to RFI were discovered, including three novel miRNAs (novel_41, novel_115, and novel_171). Only two miRNAs (novel_41 and novel_115) were expressed in all samples, indicating that most DE_miRNAs were distinct. The predicted target genes of identified DE_miRNAs are involved in a variety of cellular and molecular functions. In addition, only 6.30% of the identified common target genes were found in the liver tissue of the same subjects. These target genes primarily regulate lipid metabolism, molecular transport, intercellular communication and connections, cell death, and survival. These results provide a theoretical basis for us to understand miRNA expression profile and the molecular mechanisms of miRNA related to FE in sheep liver.

## Data Availability

The datasets presented in this study can be found in online repositories. The names of the repository/repositories and accession number(s) can be found in the article/Supplementary Material

## Supplementary material

SUPPLEMENTARY Table S1 Raw data filtering information.

SUPPLEMENTARY Table S2 Identification of all known miRNAs information.

SUPPLEMENTARY Table S3 Identification of all novel miRNAs information.

SUPPLEMENTARY Table S4 Top 20 expressed novel miRNAs information.

SUPPLEMENTARY Table S5 Top 10 miRNAs target gene prediction.

SUPPLEMENTARY FIGURE S1 Top 20 expressed known miRNAs information.

SUPPLEMENTARY FIGURE S2 Top 10 miRNAs target gene GO enrichment.
