# Supplementary figures and images for: Analysis of liver miRNA in Hu sheep with different residual feed intake
Source: Front Genet. 2023 Oct 19;14:1113411. doi: 10.3389/fgene.2023.1113411 (PMC10620975; doi:10.3389/fgene.2023.1113411)

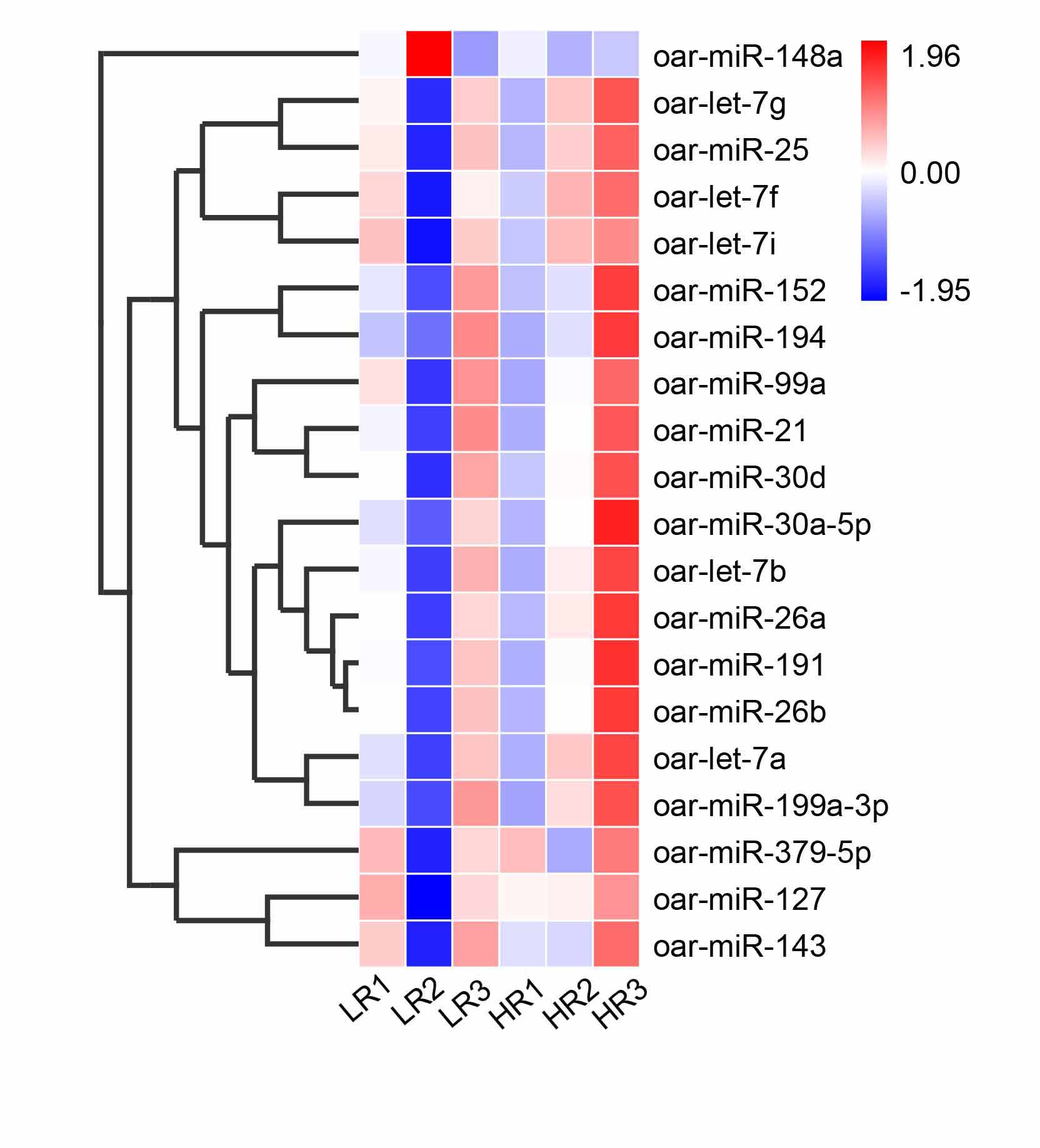

Supplement: Supplementary file 3 [file Image1.JPEG]

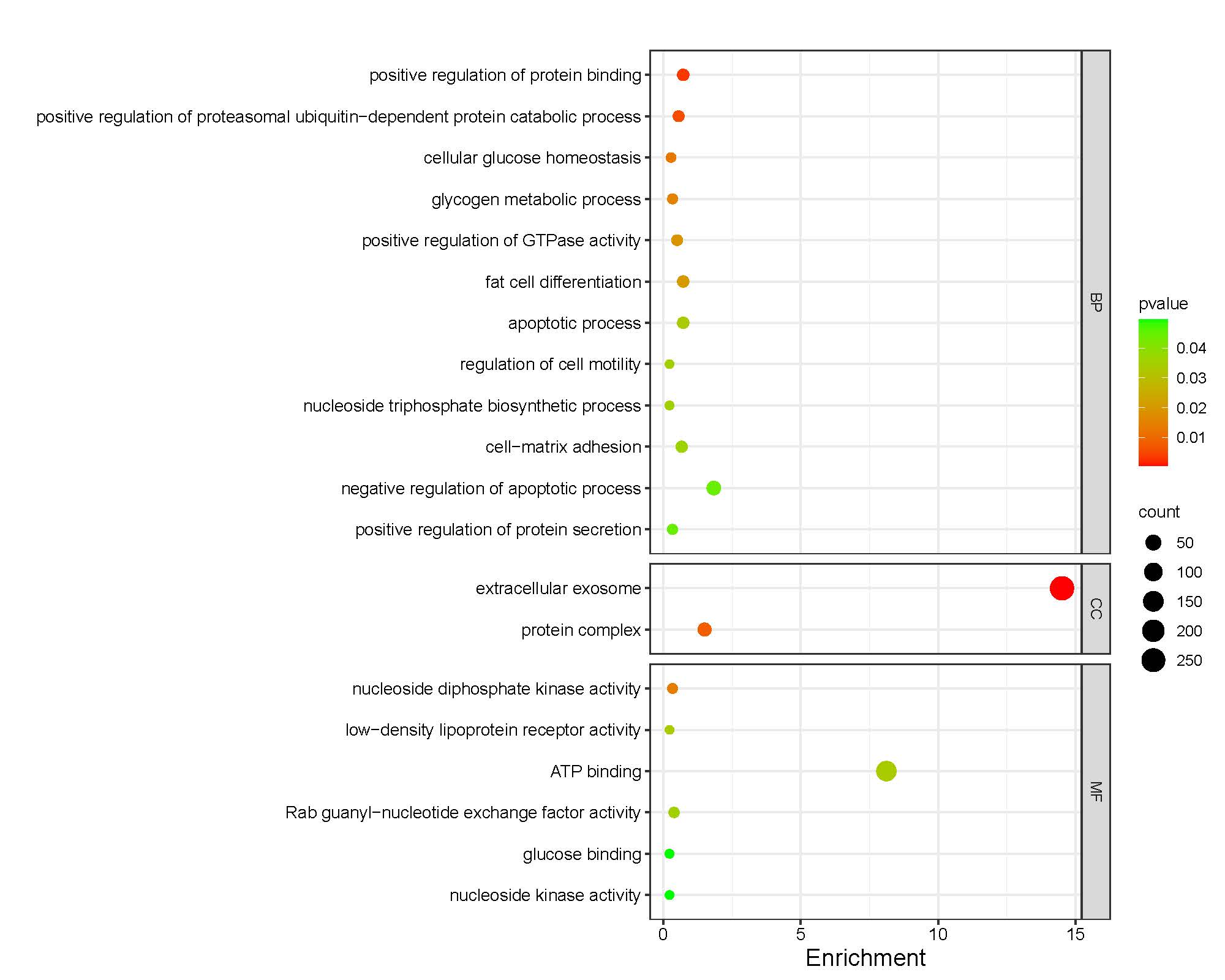

Supplement: Supplementary file 4 [file Image2.JPEG]
